# Polypharmacy in elective lumbar spinal surgery for degenerative conditions with 24-month follow-up

**DOI:** 10.1038/s41598-024-76248-6

**Published:** 2024-10-25

**Authors:** Nicholas Dietz, Chitra Kumar, Aladine A. Elsamadicy, Martin F. Bjurström, Katrina Wong, Alysha Jamieson, Mayur Sharma, Dengzhi Wang, Beatrice Ugiliweneza, Doniel Drazin, Maxwell Boakye

**Affiliations:** 1 Department of Neurosurgery, University of Louisville, 200 Abraham Flexner Hwy, Louisville, KY 40202 USA; 2 University of Cincinnati Medical School, Cincinnati, OH USA; 3 Department of Neurosurgery, Yale University, New Haven, USA; 4 Department of Surgical Sciences, Uppsala University, Uppsala, Sweden; 5 University of California, Berkeley, USA; 6 Department of Neurosurgery, University of California, San Francisco, USA; 7 Kentucky Spinal Cord Injury Research Center, University of Louisville, Louisville, KY USA; 8 Department of Neurosurgery, Providence Neuroscience Center Everett, Everett, WA USA; 9 University of Louisville, 220 Abraham Flexner Way, Louisville, KY 40202 USA

**Keywords:** Polypharmacy, Spine surgery, Health care utilization, Complications, Lumbar Fusion, Neurodegeneration, Risk factors

## Abstract

We sought to identify long-term associations of medical complications and healthcare utilization related to polypharmacy following spinal surgery for degenerative lumbar pathology. The IBM MarketScan dataset was used to select patients who underwent spinal surgery for degenerative lumbar pathology with 2-year follow-up. Regression analysis compared two matched cohorts: those with and without polypharmacy. Of 118,434 surgical patients, 68.1% met criteria for polypharmacy. In the first 30 days after discharge, surgical site infection was observed in 6% of those with polypharmacy and 4% of those without polypharmacy (*p* < 0.0001) and at least one complication was observed in 24% for the polypharmacy group and 17% for the non-polypharmacy group (*p* < 0.0001). At 24 months, patients with polypharmacy were more likely to be diagnosed with pneumonia (48% vs. 37%), urinary tract infection (26% vs. 19%), and surgical site infection (12% vs. 7%), (*p* < 0.0001). The most prescribed medication was hydrocodone (60% of patients) and more than 95% received opioids. Two years postoperatively, the polypharmacy group had tripled overall healthcare utilization payments ($30,288 vs. $9514), (*p* < 0.0001). Patients taking 5 or more medications concurrently after spinal surgery for degenerative lumbar conditions were more likely to develop medical complications, higher costs, and return to the emergency department.

**Supplementary Information:**

The online version contains supplementary material available at 10.1038/s41598-024-76248-6.

## Introduction

As many as 60–85% of older adults experience persistent musculoskeletal pain^1,2^, with 50–70% of those specifically attributed to chronic back pain^3–6^. Further, back pain is associated with functional limitation, mental health issues like depression, lower quality of life^7,8^ and is a leading cause of disability worldwide^9^. Typically, over-the-counter agents such as nonsteroidal anti-inflammatory drugs (NSAIDs) or topical analgesics are considered first-line treatments of low back pain^10^. However, patients with high pain severity are often prescribed narcotics like opioids which may provide short-term improvements in pain and function in the acute setting^11,12^. Polypharmacy, defined as the concurrent use of 5 or more medications, is associated with increased adverse events, drug-drug interactions, hospitalization and medical costs, especially in older adults^13–19^. Notably, the incidence of polypharmacy has doubled in the United States in recent years, partly due to the aging population and increased national comorbid status^19,20^. Also, certain mechanical pathologies may predispose to chronic low back pain^21^ as those with lumbar stenosis and degenerative disc disease have a higher incidence of low back pain lasting over 3 months^8,22^. Opioids and antispasmodic agents such as baclofen are commonly used for patients with chronic low back pain and spasticity, and these medications hold significant side effect profiles with risk of drug-drug interactions for older adults^23,24^.

Patients with lumbar stenosis and spondylosis requiring spine surgery are also often prescribed medications postoperatively^11^. Surgery for lumbar degenerative disorders is associated with significantly higher numbers of total drugs and pain relief medications—most frequently muscle relaxants and opioid analgesics^20^—than other surgery groups^25^.

Despite numerous studies demonstrating the adverse effects of polypharmacy^16,26^, there is limited understanding of the long-term consequences associated with polypharmacy in lumbar spine degeneration and postoperative course^27^. This study aims to address a gap in knowledge regarding specific adverse effects, hospitalization outcomes, and cost of care related to polypharmacy in patients undergoing spinal surgery for degenerative disc disease. Specifically, we compare long term sequelae of patients with spinal degeneration, including spinal stenosis, spondylosis, or disc herniation after undergoing lumbar decompression versus decompression with fusion. Primary outcomes include six-month, one-year, and two-year postoperative medical complications related to polypharmacy following spinal surgery for degenerative lumbar pathology. Secondary outcomes include categories and frequency of medications used, postoperative healthcare visits, medication refills, and cost of care.

## Methods

### Data source

Merative MarketScan Research Database, records of 2000–2021, was used for this study. MarketScan is a healthcare research insurance claims-based database that contains data for more than 265 million individuals from employer-sponsored plans. It includes data from healthcare use over time tracked with claim codes along with demographics, insurer and payments^28^. We have a neurological and neurosurgical custom database with inpatient, outpatient and prescription data. All methods were carried out in accordance with appropriate and relevant guidelines and regulations; the retrospective experimental protocol was approved by the University of Louisville Research Internal Review Board (IRB). Informed consent was waived by University of Louisville Research Internal Review Board (IRB) given the deidentified retrospective health claims database study design. Each included individual has a unique identifier that is used to link different services allowing longitudinal health services research studies.

## Patient selection

**Fig. 1:**
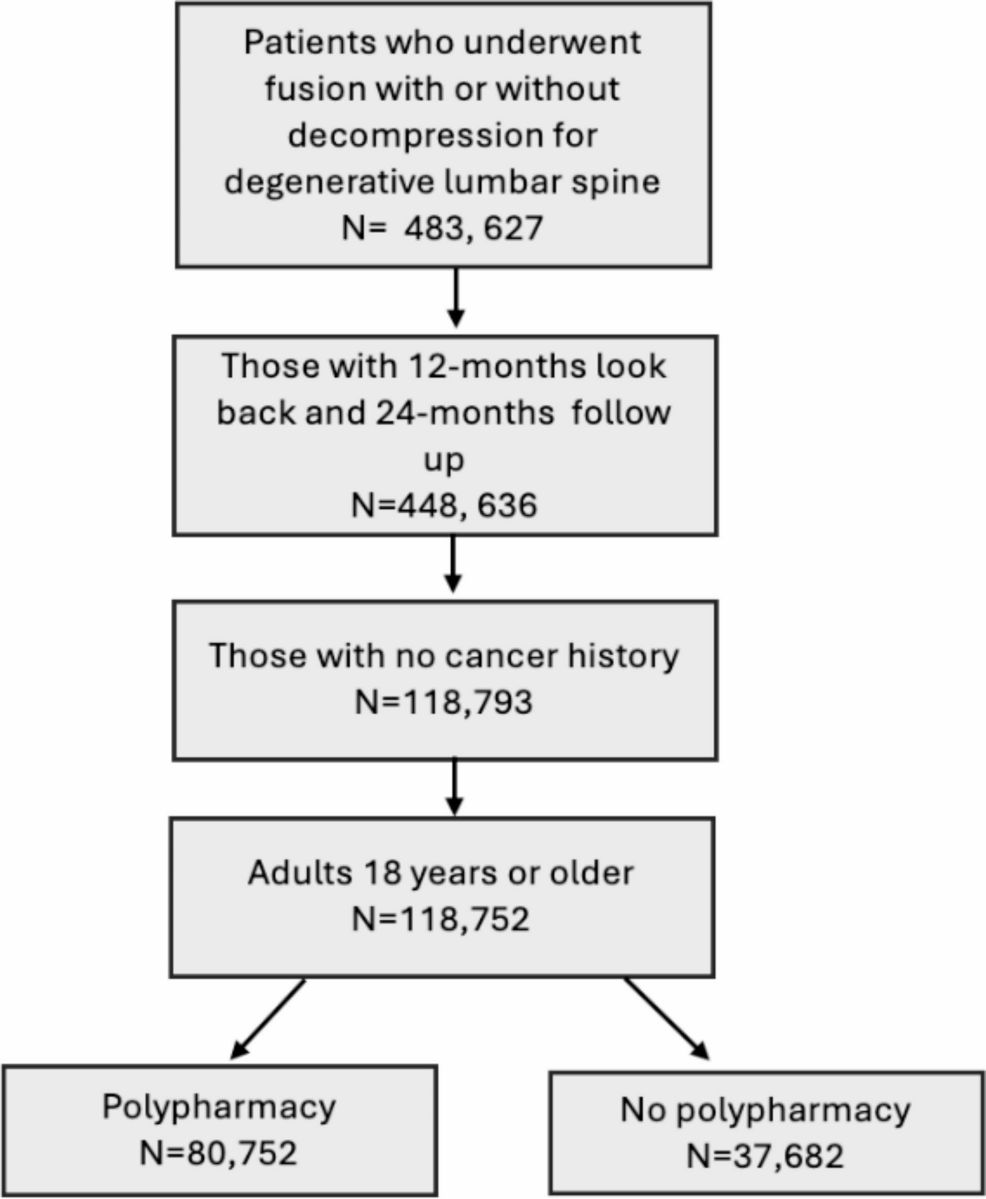
Consort Flow diagram with inclusion and exclusion criteria.

Adult patients (18 years or older) with lumbar spine degeneration (spinal stenosis, disk herniation, protrusion, and degeneration, Supplemental Table 1) who underwent surgery (fusion with or without decompression, Supplemental Table 2) were selected from inpatient admission data. International Classification of Disease 9th (prior to October 2015) and 10th revisions (October 2015 and after) and Current Procedural Terminology 4th edition (CPT-4) codes were used to identify conditions and surgical treatment. The first occurrence was set as the index hospitalization and the beginning of follow up in the data. Only those with continuous insurance enrollment for more than 1 year look back from index admission date and more than 2 years follow up from index discharge date were included. Those diagnosed with cancer in the year leading to the index hospitalization were excluded. The inclusion/exclusion process is detailed in Fig. 1.

## Pre-index look-back time and post-index follow-up time

Pre index look-back time was calculated as the difference between the insurance start enrollment date and index hospitalization admission date. If the start enrollment date was not available (0.2% of cases), the first claim date was used instead. Post index follow-up time was calculated as the difference between the index hospitalization discharge date and the insurance end enrollment date. If the end enrollment date was not available (0.3% of cases), the last claim date in the data set was used.

## Polypharmacy measure

The medications used in the follow up period (2 years post discharge from the index hospitalization) were checked to determine polypharmacy. The total number of medications used by the individuals in the included cohort was very large (> 8000), therefore, only medications in the top 75th percentile were checked. Polypharmacy was defined as the use of five or more different, concomitant medications at any time during the study period^29^. Two comparative groups were formed: polypharmacy vs. no polypharmacy. The no polypharmacy group was considered a control group.

## Patient characteristics

Patients’ characteristics include demographics (age, gender), insurance type (commercial, Medicaid or Medicare), comorbidities (Elixhauser comorbidity score obtained using used the adaptation to ICD-9-CM codes developed by Quan et al.^30^), and spine degeneration type. All patient characteristics were noted at the index hospitalization.

### Outcome of interest

We examined length of stay (LOS), total payments, and home discharge during the index hospital stay. We also evaluated hospital readmission, outpatient services, outpatient medication refills, emergency room visits, and associated payments, along with the incidence of complications (acute kidney injury, surgical site infection, cardiac arrest, deep vein thrombosis, myocardial infarction, pneumonia, pulmonary embolism, stroke, wound dehiscence, Supplemental Table 3), mental health (depression, anxiety, Supplemental Table 4), and opioid use (Supplemental Table 5) within 30 days, 6-, 12- and 24- months of index hospitalization discharge. Payments were adjusted to 2021 US dollars using the medical component of the consumer price index (accessible through the United States Bureau of Labor Statistics website, www.bls.gov.

### Statistical analysis

Continuous variables were summarized median *±* median absolute difference (MAD) as they were all found to be non-normally distributed per the Kolmogorov-Smirnov test. Categorical variables were summarized with percentages. Individual characteristics were compared using Brown Mood test for continuous variables and Chi-square test for categorical variables. To account for differences in characteristics between the 2 analysis groups, regression analysis was used to compare outcomes. *The regression models included polypharmacy (yes/no) as the test variable and all the characteristics*,* age (continuous)*,* gender (male/female)*,* insurance (commercial/Medicaid/Medicare)*,* and Elixhauser comorbidity score (0*,* 1*,* 2*,* 3 or more)*,* as control independent variables*. Quantile regression was used for continuous variables which were summarized with adjusted median *±* MAD. Logistic regression was used for categorical variables which were summarized with adjusted probabilities. To account for multiple testing, the Bonferroni^31,32^*p*-value correction was used and the significance level was set to 0.0004 (= 0.05/132 outcome comparisons). All tests were 2-sided. We used SAS 9.4 (SAS Institute, Inc, Cary, NC) for data statistical analysis.

## Results

### Patient population

A total of 118,434 patients with degenerative spine disease (Table 1) receiving spinal surgery were included.

**Table 1:** Cohort characteristics, polypharmacy is defined as having concurrently 5 or more of top 75% of medications.

Characteristics			All			Fusion with or without decompression			Decompression without fusion		
			No polypharmacy (*n* = 37,682)	Polypharmacy (*n* = 80,752)	*p*-value	No polypharmacy (*n* = 16,676)	polypharmacy (*n* = 44,411)	*p*-value	No polypharmacy (*n* = 30,909)	Polypharmacy (*n* = 64,748)	*p*-value
Age, Median ± MAD			52 ± 9	58 ± 9	< 0.0001	54 ± 9	56 ± 8	< 0.0001	52 ± 9	59 ± 9	< 0.0001
Gender, Female, n (%)			47%	55%	< 0.0001	54%	60%	< 0.0001	44%	54%	< 0.0001
Insurance	Commercial, %		71%	57%	< 0.0001	64%	60%	< 0.0001	73%	55%	< 0.0001
	Medicaid, %		15%	13%		22%	15%		12%	12%	
	Medicare, %		14%	30%		14%	25%		15%	33%	
Elixhauser index (# of comorbidities)	0, %		52%	38%	< 0.0001	43%	37%	< 0.0001	53%	38%	< 0.0001
	1, %		26%	33%		26%	31%		25%	33%	
	2, %		13%	17%		16%	18%		12%	17%	
	3+, %		10%	12%		15%	14%		9%	12%	
Specific Comorbidities	Anemia, %		8%	10%	< 0.0001	14%	14%	0.281	7%	10%	< 0.0001
	Bleeding disorders, %		1%	2%	< 0.0001	2%	2%	0.5811	1%	2%	< 0.0001
	COPD, %		6%	8%	< 0.0001	9%	8%	0.6916	6%	8%	< 0.0001
	Diabetes, %		12%	20%	< 0.0001	15%	19%	< 0.0001	11%	21%	< 0.0001
	Hypertension, %		35%	50%	< 0.0001	42%	51%	< 0.0001	34%	51%	< 0.0001
	Obesity, %		9%	9%	0.7933	11%	10%	0.0004	8%	8%	0.8113
	Morbid Obesity, %		4%	5%	< 0.0001	5%	6%	0.0112	4%	5%	< 0.0001
	Smoking, %		14%	11%	< 0.0001	16%	13%	< 0.0001	13%	10%	< 0.0001
	Weight Loss, %		0.30%	0.40%	0.0365	0.30%	0.40%	0.0941	0.30%	0.40%	0.0271
Diagnosis type	Spinal stenosis, %		30%	41%	< 0.0001	34%	39%	< 0.0001	31%	45%	< 0.0001
	Disk herniation, %		50%	34%		29%	24%		55%	37%	
	Pertusion, %		19%	24%		36%	37%		13%	17%	
	Degeneration, %		0.40%	0.60%		1%	1%		0%	1%	
Pre-surgery opioid use	# of opioids in 12 months	1–11, %	45%	63%	< 0.0001	37%	60%	< 0.0001	47%	65%	< 0.0001
		12+, %	2%	22%	< 0.0001	3%	27%	< 0.0001	2%	20%	< 0.0001
	Opioid dependent, %		0.80%	1.40%	< 0.0001	1%	2%	< 0.0001	0.60%	1.20%	< 0.0001

Of these patients, 80,752 met criteria for polypharmacy (68.1%), and 41% of those were diagnosed with spinal stenosis, 34% with disc herniation, and 24% with disc protrusion. Patients with spinal stenosis and disc protrusion were more likely to be taking 5 or more medications than those with disc herniation (*p* < 0.0001). Polypharmacy was more likely observed in older patients (median age 58 vs. 52 years, *p* < 0.0001), females (55% vs. 47%, *p* < 0.0001), and Medicare users (30% vs. 14%, *p* < 0.0001). More patients in the polypharmacy group had medical comorbidities with higher Elixhauser of 3 or more (12% vs. 10%, *p* < 0.0001). Subgroup analyses included those who received spinal fusion (*n* = 61,087) with 44,411 in the polypharmacy group and 16,676 non-polypharmacy group as well as decompression alone (*n* = 95,657) with 64,748 in the polypharmacy group an 30,909 without polypharmacy.

### Index hospitalization and discharge disposition

The regression adjusted length of hospital stay (accounting for specific comorbidities for patients with polypharmacy patients was 2.4 days and 1.4 days for non-polypharmacy patients (*p* < 0.0001), and median index hospital payments were higher for the polypharmacy group ($29,348) versus the non-polypharmacy group ($23,388) (*p* < 0.0001). Fewer patients with polypharmacy were discharged home compared to patients of the non-polypharmacy cohort (81% vs. 80%, *p* = 0.0205) (Table 2).

**Table 2:** Healthcare utilization and cost outcomes. Multivariable regression-adjusted estimates are presented. They represent the effect of polypharmacy unconfounded by observed characteristics.

Outcomes	All			Fusion with or without decompression					Decompression without fusion		
	No polypharmacy (*n* = 37,682)	polypharmacy (*n* = 80,752)	*p*-value	No polypharmacy (*n* = 16,676)	Polypharmacy (*n* = 44,411)		*p*-value		No polypharmacy (*n* = 30,909)	Polypharmacy (*n* = 64,748)	*p*-value
Index hospital											
Length of hospital stay, Median ± MAD	1.8 ± 0.5	2.4 ± 0.4	< 0.0001	3 ± 0	3 ± 0		0.125		2 ± 1	2 ± 0.5	< 0.0001
Index payment, Median ± MAD	23,388 ± 5774	29,348 ± 9264	< 0.0001	65,707 ± 4432	63,038 ± 5680		< 0.0001		19,461 ± 3447	23,564 ± 6378	< 0.0001
Discharge home, %	80%	81%	0.0205	79%	80%		0.0105		82%	83%	0.4466
6-months post-discharge											
Had 1 or more emergency visits, %	41%	53%	< 0.0001	42%	53%		< 0.0001		38%	51%	< 0.0001
Had 1 or more hospital admissions, %	13%	22%	< 0.0001	12%	20%		< 0.0001		13%	23%	< 0.0001
Number of outpatient services, Median ± MAD	17 ± 5	31 ± 6	< 0.0001	23 ± 6	35 ± 5		< 0.0001		16 ± 5	30 ± 6	< 0.0001
Number of medication refills, Median ± MAD	4 ± 2	19 ± 2	< 0.0001	3 ± 3	21 ± 2		< 0.0001		4 ± 2	19 ± 2	< 0.0001
Overall payments, Median ± MAD	2310 ± 699	6523 ± 1058	< 0.0001	3190 ± 758	6983 ± 1114		< 0.0001		2166 ± 665	6427 ± 1024	< 0.0001
12-months post-discharge											
Had 1 or more emergency visits, %	56%	68%	< 0.0001	58%	68%		< 0.0001		53%	66%	< 0.0001
Had 1 or more hospital admissions, %	23%	37%	< 0.0001	23%	34%		< 0.0001		23%	37%	< 0.0001
Number of outpatient services, Median ± MAD	31 ± 10	60 ± 11	< 0.0001	41 ± 12	67 ± 11		< 0.0001		29 ± 9	59 ± 11	< 0.0001
Number of medication refills, Median ± MAD	6 ± 3	37 ± 3	< 0.0001	6 ± 4	39 ± 4		< 0.0001		6 ± 3	36 ± 3	< 0.0001
Overall payments, Median ± MAD	4561 ± 1507	13,804 ± 2217	< 0.0001	5967 ± 1622	14,253 ± 2344		< 0.0001		4302 ± 1428	13,659 ± 2136	< 0.0001
24-months post-discharge											
Had 1 or more emergency visits, %	70%	81%	< 0.0001	69%	79%		< 0.0001		68%	80%	< 0.0001
Had 1 or more hospital admissions, %	35%	54%	< 0.0001	35%	51%		< 0.0001		34%	54%	< 0.0001
Number of outpatient services, Median ± MAD	58 ± 19	115 ± 21	< 0.0001	74 ± 22	127 ± 21		< 0.0001		54 ± 17	112 ± 20	< 0.0001
Number of medication refills, Median ± MAD	10 ± 6	71 ± 6	< 0.0001	10 ± 7	74 ± 7		< 0.0001		10 ± 6	70 ± 6	< 0.0001
Overall payments, Median ± MAD	9514 ± 3278	30,288 ± 4615	< 0.0001	12,041 ± 3619	30,907 ± 4982		< 0.0001		9097 ± 3140	30,168 ± 4460	< 0.0001

### Health outcomes and complications

Postoperative outcomes of index hospitalization, 30 days, 6, 12, and 24 months are presented in Tables 3 and 4.

**Table 3:** Complications, mental health, adverse drug events, and opioid use outcomes during index hospitalization and first 30 days.

Complications		All			Fusion with or without decompression			Decompression without fusion		
		No Polypharmacy (*n* = 37,682)	Polypharmacy (*n* = 80,752)	*p*-value	No Polypharmacy (*n* = 16,676)	Polypharmacy (*n* = 44,411)	*p*-value	No Polypharmacy (*n* = 30,909)	Polypharmacy (*n* = 64,748)	*p*-value
Index Hospital	Acute kidney injury, %	1%	1%		1%	1%		1%	1%	0.0008
	Surgical Site Infection, %	0%	1%	0.0118	0%	0%		1%	1%	
	Cardiac Arrest, %	0%	0%		0%	0%		0%	0%	
	Deep Vein Thrombosis, %	0%	0%		0%	0%		0%	0%	
	Myocardial Infarction, %	0%	0%		0%	0%		0%	0%	
	Pneumonia, %	4%	5%	< 0.0001	5%	5%		4%	5%	
	Pulmonary Embolism, %	0%	0%		0%	0%		0%	0%	
	Stroke, %	0%	0%		0%	0%		0%	0%	
	Wound Dehiscence %	0%	0%		0%	0%		0%	0%	
	Urinary Tract Infection %	2%	2%		2%	2%		2%	2%	
	At least one of the above, %	16%	19%	< 0.0001	17%	20%	< 0.0001	14%	17%	< 0.0001
1-month outcomes	Acute kidney injury, %	0%	1%	0.0219	1%	1%		0%	0%	< 0.0001
	Surgical Site Infection, %	4%	6%	< 0.0001	4%	7%	< 0.0001	4%	6%	
	Cardiac Arrest, %	0%	0%		0%	0%		0%	0%	
	Deep Vein Thrombosis, %	2%	3%	< 0.0001	2%	3%	0.0044	1%	1%	
	Myocardial Infarction, %	0%	0%		0%	0%		0%	0%	
	Pneumonia, %	6%	7%	0.0009	6%	7%	0.0379	5%	6%	0.0013
	Pulmonary Embolism, %	1%	1%		1%	1%		1%	1%	
	Stroke, %	1%	1%		0%	0%		0%	1%	0.0047
	Wound Dehiscence %	1%	2%	< 0.0001	1%	2%	0.0001	1%	2%	>0.0007
	Urinary Tract Infection %	3%	4%	< 0.0001	3%	4%	0.0038	2%	3%	< 0.0001
	At least one of the above, %	17%	24%	< 0.0001	19%	24%	< 0.0001	14%	20%	< 0.0001

**Table 4:** Complications, mental health, adverse drug events, and opioid use outcomes during index hospitalization and first 6 months, 12 months, and 24 months.

Outcomes				All			Fusion with or without decompression			Decompression without fusion		
				No Polypharmacy (n = 37682)	Polypharmacy (n = 80752)	p-value	No Polypharmacy (n = 16676)	Polypharmacy (n = 44411)	p-value	No Polypharmacy (n = 30909)	Polypharmacy (n = 64748)	p-value
6-month outcomes	Complications	Acute kidney injury, %		1%	2%	< .0001	1%	2%	0.0023	0.60%	0.90%	0.0001
		Surgical Site Infection, %		5%	8%	< .0001	6%	8%	< .0001	5%	9%	< .0001
		Cardiac Arrest, %		0%	0%	0.1052	0%	0%	0.8561	0%	0.00%	0.0417
		Deep Vein Thrombosis, %		4%	6%	< .0001	4%	6%	< .0001	2%	4%	< .0001
		Myocardial Infarction, %		1%	2%	< .0001	1%	2%	0.0129	1%	2%	< .0001
		Pneumonia, %		14%	19%	< .0001	14%	18%	< .0001	12%	17%	< .0001
		Pulmonary Embolism, %		1%	2%	< .0001	1%	2%	< .0001	1%	2%	< .0001
		Stroke, %		2%	3%	< .0001	1%	2%	< .0001	2%	3%	< .0001
		Wound Dehiscence % Urinary Tract Infection %		2% 7%	3% 9%	< .0001 < .0001	2% 12%	4% 15%	< .0001 < .0001	2% 11%	3% 15%	< .0001 < .0001
		At least one of the above, %		35%	46%	< .0001	50%	61%	< .0001	47%	60%	< .0001
	Mental Health	Depression, % Anxiety, %		16%	27%	< .0001	19%	29%	< .0001	15%	26%	< .0001
	Adverse Drug Events, n (%)			15%	23%	< .0001	16%	24%	< .0001	14%	21%	< .0001
				1%	2%	< .0001	1.60%	2.10%	0.0142	1%	2%	< .0001
	Opioid Use	# of opioids in 12 months	1–11, %	19%	26%	< .0001	18%	24%	< .0001	19%	27%	< .0001
			12+, %	10%	51%	< .0001	12%	52%	< .0001	10%	50%	< .0001
		Opioid dependent, %		5%	6%	0.0027	6%	7%	0.2378	5%	7%	0.012
12-month outcomes	Complications	Acute kidney injury, %		2%	3%	< .0001	3%	4%	0.0023	2%	3%	< .0001
		Surgical Site Infection, %		6%	10%	< .0001	6%	10%	< .0001	6%	11%	< .0001
		Cardiac Arrest, %		0.10%	0.30%	0.0089	0%	0%	0.727	0.10%	0.20%	0.0026
		Deep Vein Thrombosis, %		5%	7%	< .0001	5%	8%	< .0001	3%	5%	< .0001
		Myocardial Infarction, %		1%	2%	< .0001	1%	3%	< .0001	1%	2%	< .0001
		Pneumonia, %		23%	31%	< .0001	23%	29%	< .0001	22%	30%	< .0001
		Pulmonary Embolism, %		2%	3%	< .0001	1%	2%	< .0001	1%	3%	< .0001
		Stroke, %		3%	5%	< .0001	2%	4%	< .0001	3%	5%	< .0001
		Wound Dehiscence % Urinary Tract Infection %		2% 12%	4% 16%	< .0001 < .0001	3% 12%	5% 15%	< .0001 < .0001	2% 11%	4% 15%	< .0001 < .0001
		At least one of the above, %		49%	61%	< .0001	50%	61%	< .0001	47%	60%	< .0001
	Mental Health	Depression, % Anxiety, %		22%	36%	< .0001	26%	38%	< .0001	21%	35%	< .0001
				21%	32%	< .0001	22%	32%	< .0001	20%	30%	< .0001
	Adverse Drug Events, n (%)			3%	5%	< .0001	4%	5%	0.0001	2%	5%	< .0001
	Opioid Use	# of opioids in 12 months	1–11, %	23%	40%	< .0001	21%	37%	< .0001	24%	42%	< .0001
			12+, %	4%	34%	< .0001	5%	38%	< .0001	3%	32%	< .0001
		Opioid dependent, %		8%	11%	< .0001	10%	12%	0.017	8%	11%	< .0001
24-month outcomes	Complications	Acute kidney injury, %		6%	9%	< .0001	6%	9%	< .0001	6%	9%	< .0001
		Surgical Site Infection, %		7%	12%	< .0001	7%	11%	< .0001	7%	12%	< .0001
		Cardiac Arrest, %		0%	1%	< .0001	0%	0%	0.1033	0.20%	0.50%	< .0001
		Deep Vein Thrombosis, %		6%	10%	< .0001	7%	10%	< .0001	5%	9%	< .0001
		Myocardial Infarction, %		2%	5%	< .0001	3%	6%	< .0001	2%	5%	< .0001
		Pneumonia, %		37%	48%	< .0001	37%	46%	< .0001	35%	46%	< .0001
		Pulmonary Embolism, %		2%	4%	< .0001	3%	4%	< .0001	2%	4%	< .0001
		Stroke, %		5%	8%	< .0001	5%	7%	< .0001	5%	8%	< .0001
		Wound Dehiscence % Urinary Tract Infection %		3% 19%	4% 26%	< .0001 < .0001	3% 19%	5% 24%	< .0001 < .0001	3% 18%	4% 25%	< .0001 < .0001
		At least one of the above, %		64%	76%	< .0001	64%	74%	< .0001	63%	76%	< .0001
	Mental Health	Depression, %		31%	48%	< .0001	35%	50%	< .0001	30%	48%	< .0001
		Anxiety, %		29%	43%	< .0001	31%	43%	< .0001	28%	41%	< .0001
	Adverse Drug Events, n (%)				4%	8%	< .0001	5%	8%	< .0001	7%	< .0001
	Opioid Use	# of opioids in 12 months	1–11, %	27%	54%	< .0001	26%	52%	< .0001	26%	54%	< .0001
			12+, %	2%	25%	< .0001	2%	28%	< .0001	2%	24%	< .0001
		Opioid dependent, %		14%	20%	< .0001	17%	21%	< .0001	14%	21%	< .0001

During index hospitalization, pneumonia 5% and 4% in the non-polypharmacy group (*p* < 0.0001) and surgical site infection was present in 1% of patients with polypharmacy versus 0% in non-polypharmacy (*p* = 0.0118).

In the first 30 days after discharge, surgical site infection was observed in 6% of those with polypharmacy and 4% of those without polypharmacy (*p* < 0.0001), DVT was shown in 3% in polypharmacy and 2% of non-polypharmacy (*p* < 0.0001), urinary tract infection in 4% for the polypharmacy group versus 3% without polypharmacy (*p* < 0.0001), and acute kidney injury was found in 4% for polypharmacy and 3% in non-polypharmacy (*p* = 0.0219), Table 3. At least one complication was observed in 24% for the polypharmacy group and 17% for the non-polypharmacy group (*p* < 0.0001).

At 6 months, patients with polypharmacy were more likely to be diagnosed with pneumonia (19% vs. 14%), surgical site infection (8% vs. 5%), deep vein thrombosis (6% vs. 4%), stroke 3% vs. 2%), pulmonary embolism (2% vs. 1%), myocardial infarction (2% vs. 1%), (*p* < 0.0001), Table 4.

At 24 months, 48% with polypharmacy had pneumonia compared to 37% without polypharmacy (*p* < 0.0001), urinary tract infection was seen in 26% of those with polypharmacy versus 19% without polypharmacy, surgical site infection was seen in 12% of those with polypharmacy and 7% in the non-polypharmacy group (*p* < 0.0001), 10% with polypharmacy had DVT versus 5% without polypharmacy (*p* < 0.0001), 8% with polypharmacy had stroke versus 5% without polypharmacy, myocardial infarction was observed in 5% with polypharmacy versus 2% without polypharmacy. Depression (48% vs. 31%), anxiety (43% vs. 29%) and adverse drug events (8% vs. 4%) were also associated with those meeting criteria for polypharmacy compared with those who did not have concurrent polypharmacy at 6 months postoperatively (*p* < 0.0001). At 24 months, opioid use disorder was observed in 20% of those with polypharmacy versus 14% of those in the non-polypharmacy group (*p* < 0.0027).

### Common medication prescriptions

The most commonly prescribed medication was hydrocodone in 60% of patients, with 95% of patients receiving any opioid medication, Table 5.

**Table 5:** Top 15 drugs and classes observed in patients with polypharmacy and lumbar degenerative conditions receiving spinal surgery.

Rank	Drug name	Percent of patient use
		(*n* = 80,752)
1	APAP/HYDROCODONE BITARTRATE	60.07%
2	APAP/OXYCODONE	36.55%
3	GABAPENTIN	34.52%
4	METHYLPREDNISOLONE	30.78%
5	AZITHROMYCIN	27.23%
6	CEPHALEXIN	26.46%
7	CYCLOBENZAPRINE	25.63%
8	PREDNISONE	25.47%
9	DIAZEPAM	25.11%
10	AMOXICILLIN	24.92%
11	HYDROCODONE BITARTRATE AND ACETAMINOPHEN	22.33%
12	CIPROFLOXACIN	20.9%
13	LISINOPRIL	18.22%
14	OMEPRAZOLE	16.87%
15	METHOCARBAMOL	16.42%

Rank	Therapeutic class name	Percent of patient use
		(*n* = 80,752)
1	Anal/Antipyr, Opioid Agonists	95.05%
2	Muscle Relax, Skeletal Centra	63.92%
3	Analg/Antipyr, Nonsteroid/Antiinflam	60.84%
4	Psychother, Antidepressants	53.7%
5	Adrenals & Comb, NEC	53.13%
6	Antihyperlipidemic Drugs, NEC	53.07%
7	ASH, Benzodiazepines	44.54%
8	Anticonvulsants, Misc	43.12%
9	Gastrointestinal Drugs Misc, NEC	40.38%
10	Antibiot, Penicillins	39.67%
11	Quinolones, NEC	38.47%
12	Antibiot, Erythromycin & Macrolide	35.65%
13	Cardiac, Beta Blockers	34.26%
14	Antibiot, Cephalosporin and Rel.	34.17%
15	Cardiac, ACE Inhibitors	31.71%

Gabapentin was the third most prescribed medication at 34% followed by methylprednisolone (30%) and azithromycin (27%). Cyclobenzaprine was the most common antispasmodic agent at 25%. The second most common medication class was muscle relaxants, prescribed to 64% of patients, followed by NSAIDs (60%) and antidepressants (54%).

### Healthcare utilization and payments

Patients with polypharmacy were more likely to go to the emergency room and to be admitted at the hospital at six, twelve, and 24 months (*p* < 0.0001). Median payments for index hospital admission at time of surgery were $29,348 for those with polypharmacy and $23,388 for the non-polypharmacy group (*p* < 0.0001) with higher LOS associated with the polypharmacy group (2.4 versus 1.8 days) (*p* < 0.0001). The number of outpatient services utilized and their payments were higher for patients with polypharmacy at 6, 12, and 24 months (*p* < 0.0001). Polypharmacy patients were also more likely to request medication refills (*p* < 0.0001) and had higher prescription drug payments for all evaluated time points (*p* < 0.0001). The overall combined payments (Table 2) for inpatient and outpatient services and prescription medications six months after initial hospitalization discharge were $2,310 for non-polypharmacy patients compared to $6523 for polypharmacy patients (*p* < 0.0001). At 12 months after discharge, polypharmacy patients paid a combined median amount of $13,804 compared to $4,561 for the controls (*p* < 0.0001). 24 months after initial hospitalization discharge, polypharmacy patients paid a median combined amount of $30,288 compared to $9,514 for non-polypharmacy patients (*p* < 0.0001).

## Discussion

In the present study, 68% of patients undergoing spinal surgery for lumbar degenerative pathology met criteria for polypharmacy (taking 5 or more medications concurrently) and were more likely to incur complications such as pneumonia, urinary tract infections, surgical site infection at 6 months, 1 year, and 2 years postoperatively. Over 95% of patients were prescribed opioids (the leading medication category prescribed) and almost half were prescribed antispasmodics with cyclobenzaprine as the most commonly used at 25%. Patients meeting criteria for polypharmacy were also more likely to utilize outpatient services, visit the emergency room, and become readmitted, thereby incurring higher costs than their counterparts even 2 years post initial discharge. At two years follow-up, the polypharmacy group had tripled overall healthcare utilization payments.

Between 2000 and 2012, the incidence of polypharmacy has almost doubled in the United States^20^. According to the Center for Disease Control (CDC), 1 in 5 US adults between 40 and 79 years old used at least 5 prescription drugs in 2019^33^. The rise in polypharmacy may be attributed to the aging population, as those over the age of 65 years are more likely to be prescribed multiple medications for chronic diseases^20,34–37^. Additionally, the rise in obesity and mental health disorders likely contribute to this increasing trend^18,38^. Anxiety and depression were more likely to be observed in those taking 5 or more medications concurrently in the present study.

Polypharmacy is associated with a host of dangerous drug-drug interactions and altered pharmacodynamics and pharmacokinetics as patients age leading to adverse drug events and medical complications^24,37,39,40^. Inappropriate medication use in older adults has been reported to exceed 60%^26^. The inherent risk of adverse medical outcomes from side effect profiles and medication non-compliance^13,14,34,41,42^ has been shown to increase hospitalization and medical costs for the elderly up to 30%^15^. Opioid use was most common in those with polypharmacy following lumbar surgery, despite the trend that preoperative opioid dependence decreases after spinal surgery^43–45^.

It is likely that patients with polypharmacy have poorer health status at baseline or post-injury and are disproportionally at risk of medical complications. However, despite regression-controlled analyses for comorbidities we saw increased risk of postoperative complications. One of the largest discrepancies for the polypharmacy cohort was observed in relation to postoperative pneumonia. Opioids and benzodiazepines are associated with increased risk of pneumonia secondary to immunosuppressive effects and possibly respiratory depression^46^. Dublin and colleagues report that the odds for pneumonia was more than 200% higher (OR 3.24) in individuals newly prescribed opioids and almost 100% higher (OR 1.88) regardless of time of use^46^. Additionally, surgical site infection during index hospitalization, the first 30 days post-discharge, and a seemingly compounding effect across 2 years. SSI is associated with 3–5% of lumbar surgery cases^47^ and higher healthcare utilization^48,49^. Many studies have shown that smoking, obesity, diabetes, and hypertension represent significant risk factors for development of SSI^50^. Polypharmacy may also represent an indirect risk factor to signal to clinicians an increased risk associated with early and late SSI.

Chilakapati et al. showed that preoperative polypharmacy was associated with increased readmission within 90-days of a corrective spinal deformity surgery in adults^51^. Older adults in the polypharmacy cohort had a higher rate of readmission within the 90-day window. Our study extends the follow up timeline and demonstrates an increased risk of readmission even at 2 years post-operation. Sato et al. investigated 767 patients 65 or older retrospectively who underwent knee arthroplasty, hip arthroplasty or spine surgery for degenerative conditions^25^ and found that greater than 50% of these patients were taking 6 or more prescription medications.

Despite a scarcity of literature assessing the long-term effects of polypharmacy in spinal degenerative surgery, there is relatively more research in other surgical fields on the effects of polypharmacy. In 2016, Harstedt et al. found that polypharmacy was predictive of rehospitalization in a case review of 272 patients with hip fracture who underwent acute total hip arthroplasty^17^. Similarly, Holden et al. found that patients over the age of 60 undergoing bilateral transfer abdominus release for ventral hernias were more likely to suffer complications, postoperative delirium, increased hospital LOS, and cardiac events if they engaged in polypharmacy^52^. In a retrospective series of 584 patients who underwent abdominal surgery, Abe et al. found that polypharmacy was a strong predictive factor for prolonged hospitalization^53^. Arends et al. investigated 518 patients above the age of 70 undergoing cardiac surgery to analyze the association between preoperative medication use and functional decline post-surgery^54^. They found that preoperative polypharmacy was associated with higher risks of functional decline (defined as either disability or a decreased health-related quality of life) after cardiac surgery. Our results corroborate these findings of increased complications associated with polypharmacy postoperatively following spine surgery. In 2020, Cadel et al. conducted a systematic review on polypharmacy in patients with spinal cord injuries^27^ and found that negative clinical outcomes such as drug-related issues and bowel complications were associated with polypharmacy^27^. Kitzman et al. designed a retrospective case-control study in 2016 to analyze the association of polypharmacy and spinal cord injury^29^ and found that patients with spinal cord injuries were prescribed multiple medications and most from drugs with high rates of toxicity or adverse effects. Our analysis found that polypharmacy in spinal degeneration was associated with more postoperative complications.

Nazemi et al. published a literature review in 2017, evaluating studies and systematic reviews published between 1990 and 2015, to create an algorithm for preventing and managing delirium in geriatric patients who undergo elective spinal surgery^55^. They found that polypharmacy is an independent risk factor for delirium which can increase length of hospital stay to greater than 7 days. Polypharmacy is a well-described risk factor for delirium, especially in older adults^56,57^. Additionally, other studies have described an increased risk of dementia diagnosis—another risk factor for delirium—following spinal surgery with associated increased healthcare utilization^58,59^. However, a prospective study conducted on 250 patients with an average age of 72 years in Thailand demonstrated that there was no significant association between polypharmacy and post-operative cognitive decline^60^.

### Strengths and limitations

The degree to which polypharmacy contributed to increased risk of medical complication in patients with likely higher degree of pre-existing medical comorbidities is uncertain. We cannot conclude a causal relationship of polypharmacy to complications observed. However, a strength of the analysis is the large sample size that demonstrates clinical trends across populations. One limitation of this study includes consistent charting of “polypharmacy” with multiple accepted definitions in the literature. The definition of polypharmacy varies by providers and authors, and does not always specify whether the discussion is limited to prescription or non-prescription drugs only and what specific categories those drugs fall into, for example. Future studies may specifically analyze categories of drugs and how they affect long-term outcomes^61^. Another limitation includes the use of paid claims data such that variation in diagnosis and severity of postoperative complications may be reported. As other claims databases, results should be interpreted and generalized cautiously especially considering that billing codes are also prone to human error. Even so, the MarketScan Research Database allows users to follow patients long-term and appreciate their postoperative course and quality of life. Future studies may also examine ways in which enhanced recovery after surgery (ERAS) protocols with multimodal pain management strategies may reduce risk of postoperative polypharmacy^62^.

## Conclusion

Opioids were the most commonly prescribed drugs for those meeting criteria of polypharmacy, observed in more than 95% of patients. Notably, pneumonia, urinary tract infections, and surgical site infection were observed at higher rates postoperatively for those with polypharmacy that increased across 24 months. Patients taking 5 or more medications concurrently after spinal surgery for degenerative lumbar conditions were more likely to develop medical complications across two years after surgery and return to the emergency department and utilize more outpatient services than non-polypharmacy counterparts.

## Electronic supplementary material

Below is the link to the electronic supplementary material.

Supplementary Material 1

Supplementary Material 2

Supplementary Material 3

Supplementary Material 4

Supplementary Material 5

## Data Availability

All data generated or analyzed during this study are included in this published article and its supplementary information files.
