# Supplementary material for: Polypharmacy in elective lumbar spinal surgery for degenerative conditions with 24-month follow-up
Source: Sci Rep. 2024 Oct 25;14:25340. doi: 10.1038/s41598-024-76248-6 (PMC11511981; doi:10.1038/s41598-024-76248-6)
Supplement: Supplementary file 1 — Supplementary Material 1 [file 41598_2024_76248_MOESM1_ESM.docx]

**Supplemental Table 1**: ICD-9 and ICD-10 codes for lumbar spine degeneration diagnoses included.

| **Diagnostic** | **ICD-9 Code** | **ICD-10 Code** |
| --- | --- | --- |
| spinal stenosis | 724.02 | M48.06, M48.07, M99.23- M99.73 |
|  | 724.03 -caudication |  |
| disk herniation | 722.10 | M51.26, M51.27 |
| pertusion | 722.32 | M51.46, M51.47 |
|  | 722.52 | M51.36, M51.37 |
|  | 722.73 | M51.06, M51.07 |
|  | 722.93 | M51.86, M51.87 |
| degenerative | 724.3 | M54.3 |
| conditions | 724.2 | M54.4 |
