# Supplementary material for: Polypharmacy in elective lumbar spinal surgery for degenerative conditions with 24-month follow-up
Source: Sci Rep. 2024 Oct 25;14:25340. doi: 10.1038/s41598-024-76248-6 (PMC11511981; doi:10.1038/s41598-024-76248-6)
Supplement: Supplementary file 2 — Supplementary Material 2 [file 41598_2024_76248_MOESM2_ESM.docx]

**Supplemental Table 2**: ICD-9, ICD-10, and CPT-4 codes for lumbar spine degeneration diagnoses included.

| **Procedures** | **ICD-9 Code** | **ICD-10 Code** | **CPT Code** |
| --- | --- | --- | --- |
| **Decompression** | 03.09 | 0RB*xy*ZZ(*x*=0,1,4,6,A) - Excision | 63001, 63003, 63005, 63011, 63012, 63015, 63016, 63017, 63040, 63041, 63042, 63045, 63046, 63047 |
|  |  | 0SBxyZZ(x=0,3) - Excision |  |
|  |  | 00N*xy*ZZ(*x*=W,X,Y) -Release | 63005, 63012, 63017,63030, 63035, |
|  |  | 009*xy*0Z - Drainage |  |
|  |  | 009xyZZ(*x*=T,W,X,Y) - Drainage |  |
|  |  | 009U00Z, 009U0ZZ - Drainage |  |
|  |  | 00JV0ZZ, 00JU0ZZ -Inspection |  |
| **Lumbar Fusion** | 81.06, 81.07, 81.08 | 0SG0, 0SG1, 0SG3 | 22533, 22558, 22586, 22612, 22630, 22633 |
