# Supplementary material for: Polypharmacy in elective lumbar spinal surgery for degenerative conditions with 24-month follow-up
Source: Sci Rep. 2024 Oct 25;14:25340. doi: 10.1038/s41598-024-76248-6 (PMC11511981; doi:10.1038/s41598-024-76248-6)
Supplement: Supplementary file 3 — Supplementary Material 3 [file 41598_2024_76248_MOESM3_ESM.docx]

**Supplemental Table 3**: ICD-9 and ICD-10 code for complications evaluated.

| **Complications** | **ICD9** | **ICD10** | **CPT-4** |
| --- | --- | --- | --- |
| Acute Kidney Injury | 584.5, 584.6, 584.7, 584.8, 584.9 | N17 |  |
| Any surgical site infection | 996.66, 996.67, 998.51,998.59, 998.83 | T84.5*xx*A, T84.6*xx*A, T84.7*xx*A, T81.4*xx*A, K68.11 |  |
| Cardiac Arrest | 427.5, 427.41, 427.5, 785.51, | I46.9, I49.01, I46, R57.0, | 92950 |
| Deep vein thrombosis | 453.2, 453.3, 453.4, 453.8 | I82.2*x*0, I82.3, I82.4, I826, I82.890, I82.90, I82.A1, I82.B1, I82.C1 |  |
| Myocardial Infarction | 410.*x*0, 410.*x*1 | I21 |  |
| Pneumonia | 480,481, 482,483, 484, 485, 486 | J12, J13, J14, J15, J16, J17, J18 |  |
| Pulmonary Embolism | 415.1 | I26.9 |  |
| Stroke | 997.01 ,997.02, 433.*x*1, 434.*x*1 | G97.81, G97.82, I97.81, I97.82, I63 |  |
| Wound Dehiscence | 998.30, 998.31, 998.32 | T81.30*x*A, T81.31*x*A, T81.32*x*A |  |
