# Supplementary material for: Polypharmacy in elective lumbar spinal surgery for degenerative conditions with 24-month follow-up
Source: Sci Rep. 2024 Oct 25;14:25340. doi: 10.1038/s41598-024-76248-6 (PMC11511981; doi:10.1038/s41598-024-76248-6)
Supplement: Supplementary file 4 — Supplementary Material 4 [file 41598_2024_76248_MOESM4_ESM.docx]

**Supplemental Table 4**: ICD-9 and ICD-10 codes for mental health conditions evaluated

| **Mental Health** | **ICD9** | **ICD10** |
| --- | --- | --- |
| Major Depressive Disorder | 296.2, 296.3 | F32.0-F32.5, F32.9, F33.0-F33.4, F33.9 |
| Other Depression | 296.82, 311 | F32.8, F33.8 |
| Anxiety | 300.00-300.02, 300.09, 300.21-300.23 | F40.0, F40.1, F41.0, F41.1, F41.8, F41.9 |
