# Supplementary material for: Polypharmacy in elective lumbar spinal surgery for degenerative conditions with 24-month follow-up
Source: Sci Rep. 2024 Oct 25;14:25340. doi: 10.1038/s41598-024-76248-6 (PMC11511981; doi:10.1038/s41598-024-76248-6)
Supplement: Supplementary file 5 — Supplementary Material 5 [file 41598_2024_76248_MOESM5_ESM.docx]

**Supplemental Table 5**: Opioid prescription drug names, ICD-9 and ICD-10 for Opioid use Disorder

| Opioid Use Disorder | ICD-9 | 304.00-304.02, 305.50-305.52 |
| --- | --- | --- |
|  | ICD-10 | F11 |
| Opioid Prescription drugs | Buprenorphine, Butorphanol, Codeine, Dihydrocodeine, Fentanyl, Hydrocodone, Hydromorphone, Levorphanol, Meperidine, Methadone, Morphine, Nalbuphine, Opium, Oxycodone, Oxymorphone, Pentazocine, Propoxyphene, Tapentadol, Tramadol, Buprenorphine, Naltrexone | |
